# Portal vein embolization with n-butyl-cyanoacrylate through an ipsilateral approach before major hepatectomy: single center analysis of 50 consecutive patients

**DOI:** 10.1186/s40644-017-0127-3

**Published:** 2017-09-20

**Authors:** José Hugo Mendes Luz, Paula Mendes Luz, Tiago Bilhim, Henrique Salas Martin, Hugo Rodrigues Gouveia, Élia Coimbra, Filipe Veloso Gomes, Roberto Romulo Souza, Igor Murad Faria, Tiago Nepomuceno de Miranda

**Affiliations:** 1 Department of Interventional Radiology, Radiology Division, National Cancer Institute, INCA, Praça Cruz Vermelha 23, Centro, Rio de Janeiro, CEP 20230-130 Brazil; 2 National Institute of Infectious Disease EvandroChagas, Oswaldo Cruz Foundation, Rio de Janeiro, Brazil; 3 Department of Interventional Radiology, Centro Hepato-Bilio-Pancreático e de Transplantação.Hospital Curry Cabral, CHLC, Lisbon, Portugal

**Keywords:** Portal vein, Embolization, Future liver remnant, Extended hepatectomy, Hepatic insufficiency

## Abstract

**Purpose:**

To evaluate the efficacy of portal vein embolization (PVE) with n-Butyl-cyanoacrylate (NBCA) through an ipsilateral approach before major hepatectomy. Secondary end-points were PVE safety, liver resection and patient outcome.

**Methods:**

Over a 5-year period 50 non-cirrhotic consecutive patients were included with primary or secondary liver cancer treatable by hepatectomy with a liver remnant (FLR) volume less than 25% or less than 40% in diseased livers.

**Results:**

There were 37 men and 13 women with a mean age of 57 years. Colorectal liver metastases were the most frequent tumor and patients were previously exposed to chemotherapy. FLR increased from 422 ml to 629 ml (*P* < 0.001) after PVE, corresponding to anincrease of 52%. The FLR ratio increased from 29.6% to 42.3% (P < 0.001). Kinetic growth rate was 2.98%/week. A negative association was observed between increase in the FLR and FLR ratio and FLR volume before PVE (*P* = 0.002). In 31 patients hepatectomy was accomplished and only one patient presented with liver insufficiency within 30 days after surgery.

**Conclusions:**

PVE with NBCA through an ipsilateral puncture is effective before major hepatectomy. Meticulous attention is needed especially near the end of the embolization procedure to avoid complications.

**Trial registration:**

Clinical Study ISRCTN registration number: ISRCTN39855523. Registered March 13th 2017.

## Background

More than 30 years after its first publication, portal vein embolization (PVE) is still abundantly used to successfully promote hepatic hypertrophy before major hepatectomies [1]. Hepatic resection is currently the cornerstonein the curative treatment of primary liver malignancies such as cholangiocarcinoma, hepatocellular carcinoma and metastasis from colorectal cancer and other primary origins [2]. To allow hepatic resection most hepatobiliary services admit a future liver remnant (FLR) of at least 25% in healthy livers [3]. For diseased livers, as in heavily chemotherapy treated patients [4, 5]or in hepatic cirrhosis candidates [6], larger FLR of 35% to 40% are required. Embolization of the aimed portal vein territory will diverge all blood flow containing trophic and growth factors to the FLR, inducing hypertrophy and permitting the prearranged future surgery [7]. As with PVE, liver surgery greatly evolved over the years allowing more patients to undergo this potentially curative treatment [8, 9]. Systemic chemotherapy additionally played an important role downsizing tumors and converting previously unresectable patients into surgical candidates [10]. PVE has been shown to besafe and effective in promoting FLR growth [3, 11] and is currently adopted in the preoperative scenario in many hepatobiliary units worldwide [12].

A myriad of technical approaches and different embolic materials have been proposed [13]. To date, n-butyl-cyanoacrylate (NBCA) has been used for PVE and some publications have suggested that it may be more efficient than other embolic agents [14]. PVE with NBCA has been widely adopted throughout the last 3 decades. However, the percutaneous access has been nearly exclusively through the FLR as originally described in France [4, 15].Nevertheless, access to the portal vein is usually through the diseased liver that is going to be surgically removed (ipsilateral side) when using other embolic agents, such as particle embolics and coils [16–20]. Furthermore, in situations that PVE with NBCA was attempted through the ipsilateral side authors have done it with the aid of either amplatzer plugs [21, 22] or occlusion balloons [23] in order to minimize the risk of NBCA reflux to FLR. We conducted the present study to assessthe efficacy and safety of PVE solely with NBCA through an ipsilateral approach.

## Methods

### Patients

Over a 5-year period 50 consecutive patients with primary and secondary liver cancer referred for PVE before major hepatectomy were assessed for analysis. Inclusion criteria were: patients with primary and secondary liver cancer treatable by hepatectomy with a proportion of FLR volume to the total functional liver volume (TFLV) less than 25% orless than 40% in patients with previous chemotherapy or hepatic cirrhosis. Exclusion criteria were: extensive ipsilateral tumor precluding safe access to the portal vein, unmanageable coagulopathy, extensive extra-hepatic disease, liver abscess or infection. PVE indications and details, including embolization of segment IV, were discussed and decided previously in the weekly multidisciplinary liver tumor board meeting. All patients gave their written informed consent to be submitted to PVE. The ethics committee of the Brazilian’s National Cancer Institute (INCA) approved the study protocol (Approval #67703317.1.0000.5274). The clinical and imaging records from these sequential patients were retrospectively gathered from the hospital archive and liver volumetric data was generated as stated in the liver volume section.

### Study endpoints

Primary endpoint was to evaluate the efficacy of PVE with the NBCA through an ipsilateral approach. Secondary end-points were accomplishment of liver surgery, patient out-come after hepatectomy and safety of the proposed PVE technique. Efficacy was measured according to FLR volume changes, growth rate and kinetic growth rate and was obtained from 37 out of the 50 patients due to unavailable full imaging follow-up data (pre or post-PVE complete set of imaging studies) in 13 patients. All other analysis refers to the total study population (50 patients).

### Portal vein Embolization

On the day of PVE patients were assigned to a hospital bed with an anticipated 24 h hospitalization. Patients were kept on intravenously conscious sedation (*n* = 33) or general anesthesia (*n* = 17) depending on patient collaboration and anesthesiologist preference. Except for the side which we decided to puncture the liver, our PVE technique was accomplished similarly as reported elsewhere [4, 15]. In brief, a non-FLR portal branch was punctured through ultrasound guidance always avoiding tumor transgression. A 6-F vascular sheath (Terumo, Tokyo, Japan) was placed in the portal vein branch accessed and a subtraction acquisition was performed through a 5F pigtail angiographic catheter (Cook Medical, Bloomington, IN). Selective catheterization of each second-order portal vein branches was achieved with 5F Simmons 1 or 2 catheters (Cook Medical) in the first 30 patients. Coaxial microcatheters (2.8-F Progreat, Terumo) were additionally used in the last 20 patients. Small boluses of n-butyl-cyanocrylate (NBCA - Hystoacryl^®^, Trudell Medical International, London, Canada) with iodized oil (Lipiodol^®^ Guerbet, France) in a ratio that varied from1-to-3 to 1-to-5 depending on the specific portal vein branch flow, flushed with 5% dextrose, was used for embolization. Segment IV embolization was also completed with glue (*n* = 6) except in technically defiant or very small branches in which polyvinyl alcohol microparticles (100-300 μm Beadblock, Biocompatibles, Farnham, UK) were used (*n* = 4) as suggested in previous publications [24]. A post-embolization direct portography was obtained and the glue cast image was recorded. Liver parenchymal tract occlusion was performed with the NBCA lipiodol mixture. Intravenous prophylactic antibiotics were administered at the moment of PVE. After hospital discharge patients were kept on oral analgesics as needed.

### Liver volumetry

A 3.0 mmor less slice thickness CT was obtained during the arterial and portal phases with a 16-detector row multislice CT scanner (Phillips, The Netherlands). On individual slices the whole liver, the tumor and the FLR (accordingly to previously surgical planning) were delineated with a handheld cursor using a freely downloadable open-source image analysis software package, OsiriX^**®**^. This open-source PAC software system was agreed to be used since it has been reported and validated for liver volumetric assessment elsewhere [25]. Once all of the regions of interest were selected within one series, the volumetric calculations were obtained using OsiriX^**®**^ by multiplying surface and slice thickness and then adding up individual slice volumes [25]. TFLV comprehended the total hepatic volume subtracted by the tumor volume. FLR was defined as the portion of the liver that would remain after the proposed hepatectomy. The ratio between the FLR and the TFLV was calculated and defined as the FLR/TFLV ratio. The increase in the FLR after PVE was also quantified and calculated by the formula ((FLR post PVE - FLR pre PVE) ÷ FLR pre PVE) as suggested in guidelines [26]. Additionally, the kinetic growth rate (KGR), defined as the increase in the FLR/TFLV ratio divided by the length of time (in weeks) was calculated [27].

### Complications and patient outcome

Pain during and after PVE was assessed with 10-point pain scales. Complications were obtained from the clinical, imaging and laboratory data files and from PVE reports. Complications were classified as suggested in previous publications [15, 28]. Major complications were defined as events that promoted significant morbidity raising the level of medical treatments, or that prolonged hospitalization or provoked hospital re-admissions. Events that did not promote longer hospitalization or did not require specific treatment were considered as incidental findings (e.g., migration of minimal NBCA fragments in the FLR) [15]. Liver enzymes and liver function were assessed before PVE, before surgery and in the immediate postoperative scenario. Patients’ charts were scrutinized for submission to surgery, reasons for precluding surgery, surgical complications, intensive care unit admissions, transfusions, length of hospital permanence and death. For all 50 patients included in this study, medical reports were analyzed to the most updated available information up to December 2016 or death. The mean follow-up time was 23.5 months (range 1-60, SD 19.22).

### Statistical analysis

Descriptive statistics including mean, standard deviation and range were calculated for numerical variables while absolute numbers and percentages were calculated for categorical variables. Comparison of TFLV and FLR volumes before and after PVE were performed by either paired t-test or paired Wilcoxon rank-sum test, as appropriate. Linear regression models were used to test the association between FLR volume before PVE and FLR volume increase after PVE and between FLR/TFLV ratio before PVE and FLR volume increase after PVE. The association between the use of microcatheter and the occurrence of complications was tested using Fisher’s exact test and Chi-squared test.

## Results

**Fig. 1:**
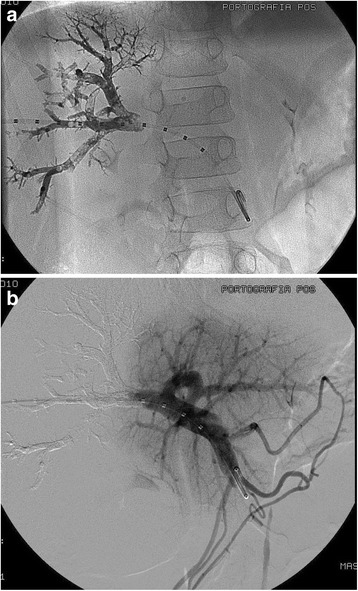
**a** Glue cast at the end of PVE. Glue cast at the end of PVE in an 8 year-old boy with right-liver Hepatoblastoma showing satisfactory NBCA deposition in the right portal branches. **b** Post-embolization direct portography. Post-embolization direct portography in the same patient showing occlusion of the right portal branches and good flow to the left portal vein

There were 37(74%) men and 13(26%) women with a mean age of 57 years ±15 (range, 5–80 years). Colorectal liver metastases were the most frequent tumor (Table 1). All patients with colorectal cancer had been previously exposed to systemic chemotherapy. No patients presented with liver cirrhosis, including the ones with hepatocellular carcinoma. Four patients showed biliary obstruction at presentation and were percutaneously drained before (*n* = 3) or at the moment (*n* = 1) of PVE.In 49 (98%) patients the ipsilateral approach was performed while in 1 patient both ipsilateral and contra-lateralside punctures were performed. Mean pain score during and after the procedure was 2.5±2.5 points. Mean hospital stay was 1.1 days. Thirty-eight (76%) patients had a right PVE,10 (20%) patients had a right PVE plus segment IV and 2 patients underwent PVE of segments VI and VII (4%), (Fig. 1).

**Table 1:** Patients’ characteristics

Number of patients	50
Age, mean (SD)	56.5(15.1)
Male patients, N (%)	37 (74)
Tumor, N (%)	
Cholangiocarcinoma	7 (14)
Colorectal	36 (72)
HCC	3 (6)
Hepatoblastoma	2 (4)
Metastases Wilms Tumor	1 (2)
Retroperitoneal Leiomyiosarcoma	1 (2)
Chemotherapy, N (%)	39 (78)
Biliary drainage, N (%)	4 (8)
Arterial embolization, N (%)	3 (6)
Ablation before PVE, N (%)	4 (8)

*SD* Standard Deviation

PVE was technically successful in 49 (98%) patients. Assisted secondary technical success was obtained in all 50 patients. PVE was technically incomplete in 1 patient as it was necessary to repeat the procedure to achieve full occlusion of an anterior sectorial branch that was overlooked. Segment IV embolizations were carried out by the ipsilateral approach in all but one patient. This patient was submitted to a second PVE to occlude segment IV branches as decided in the tumor board meeting a few days after the completion of the first PVE procedure. Since all right portal vein branches were already occluded we were obligated to perform segment IV embolization through the contralateral approach. In this case it was necessary to puncture the FLR, which occurred uneventfully.

Regarding biliary obstruction; in 3 patients we obtained a significant reduction of bilirubin levels after drainage, before performing PVE. In 1 patient the biliary drainage was accomplished at the same moment of PVE. For this patient we performed PVE followed by biliary drainage at the same procedure. This tactic was implemented to try to optimize the time gap between PVE and surgery, as it is suggested in some publications [29]. Currently we first obtain an adequate biliary decompression with undoubtful evidence of declining levels of bilirubin before we proceed to PVE in such subset of patients [30].

### Volumetric liver results and laboratory values

**Fig. 2:**
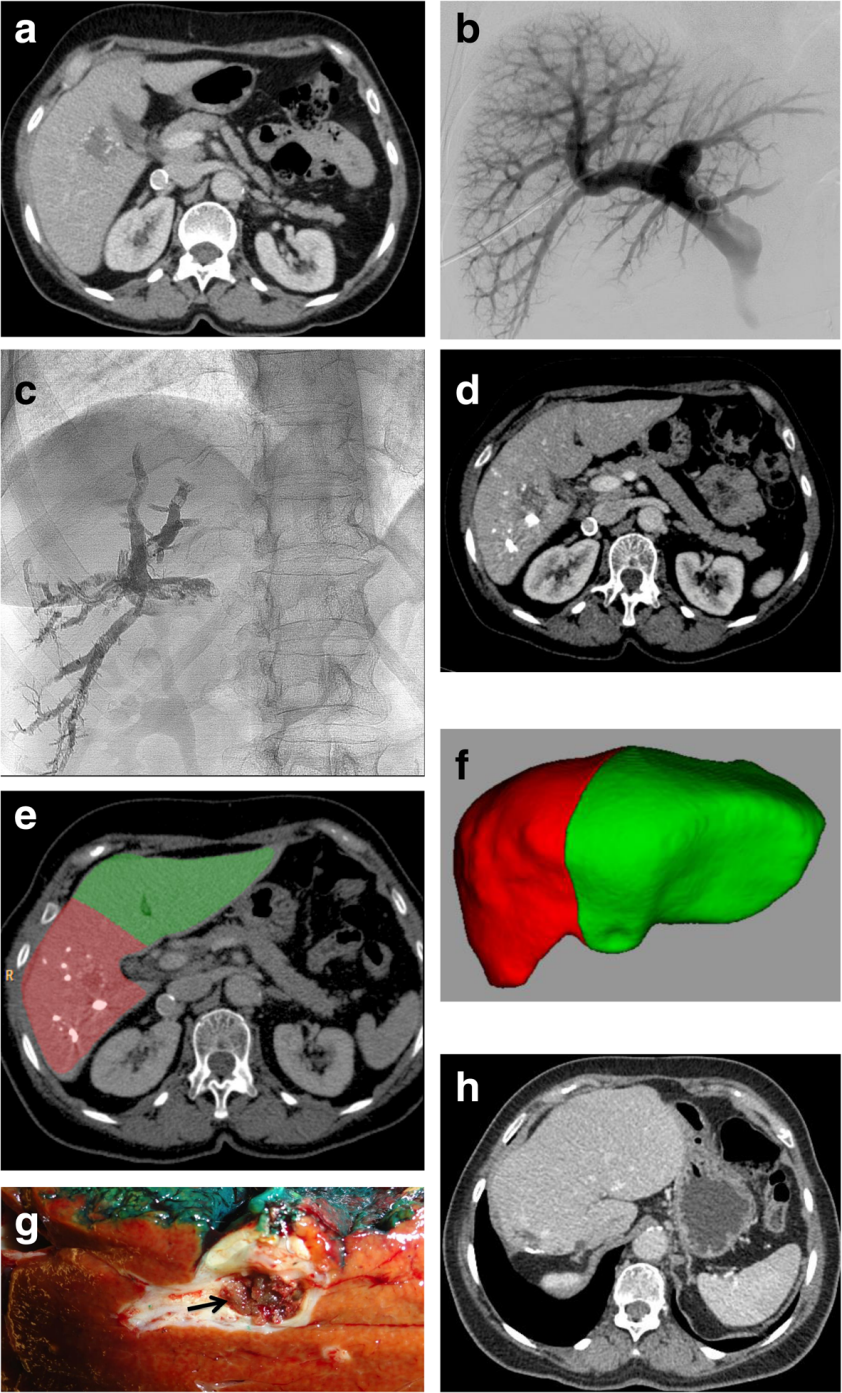
**a** Computed tomography before PVE. A contrasted portal phase computed tomography before PVE in a 67 year-old female with colorectal cancer and liver metastasis. **b** Direct portography. Direct portography depicting normal portal vein anatomy during PVE. **c** Glue cast. Glue cast in the right portal branches at the end of PVE showing satisfactory distribution of the NBCA-lipiodol mixture. **d** Computed tomography 30 days after PVE. Portal venous phase computed tomography 30 days after PVE showing an important hypertrophy of the left liver. **e** and **f** Computed tomography volumetry after PVE. Computed tomography volumetry yielded a FLR increase of 44% and a FLR/TFLV ratio expansion from 34% to 47% after 30 days. **g** Liver specimen after right hepatectomy. Liver specimen after right hepatectomy showing glue in a right portal vein branch from the previous portal vein embolization. **h** 3-year post-operative computed tomography. Post-operative portal venous phase computed tomography 3 years after PVE with a good remnant liver volume

**Fig. 3:**
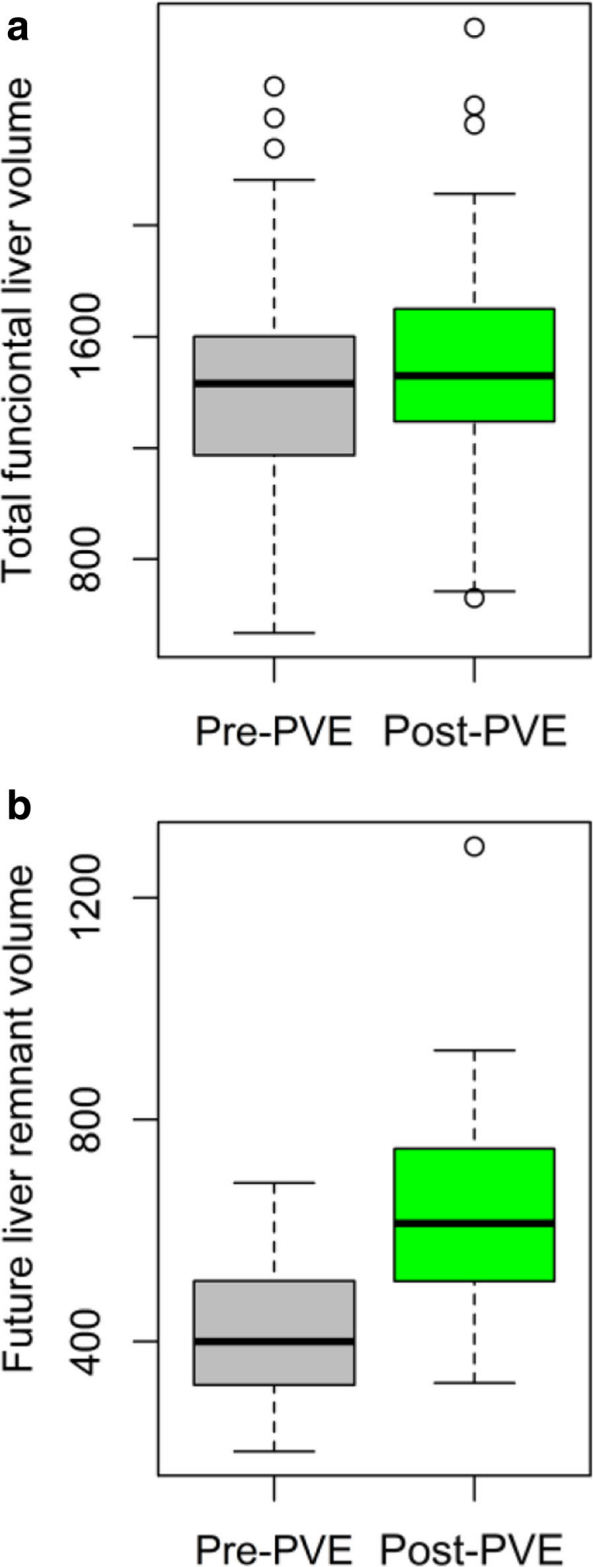
Graph showing increase in TFLV and FLR volume. Graph showing slight increase in the TFLV after PVE (top graph) and significant increase in FLR volume 1 month after PVE using NBCA (*P* < 0.001 – *bottom graph*)

CT assessments were performed on average 15 (range 1–22) days before PVE. Imaging interval from the day of PVE to the post procedure volumetric CT was 32.7 ±14.5 days. FLR increased froma mean value of 422 ml ±133 to 629 ml ±192 (*P* < 0.001) after PVE, corresponding to a mean FLR increase of 52%±22% (Table 2, Figs. 2 and 3). The FLR/TFLV ratio increased from 29.6% ± 8.3% to 42.3% ± 9.8% (P < 0.001). Kinetic growth rate was 2.98%/week ± 1.29%/week. The TFLV slightly increased from 1474 ± 433 to 1531 ± 460 after PVE did not reach statistical significance (*P* = 0.070). Laboratory values showed no significant changes of measured parameters at 4–5 weeks after PVE when compared with measurements before PVE (Table 2).

**Table 2:** Liver volumetry before and after PVE

PVE segments, N (%)	
Right plus IV PVE	10 (20)
Right PVE	38 (76)
Segments VI and VII	2 (4)
PVE approach, N (%)	
Ipsilateral	49 (98)
Ipsi and Contra-lateral	1 (2)
Microcathether, N (%)	20 (40)
Glue: Lipidol ratio (range)	1-3 to 1-4
Before PVE^a^	
Total functional liver volume, mL, mean (SD)	1473.57 (432.78)
Future liver remnant, mL, mean (SD)	421.95 (132.54)
After PVE^a^	
Total functional liver volume, mL, mean (SD)	1531.24 (459.77)
Future liver remnant, mL, mean (SD)	628.97 (191.64)
FLR increase^a^, %	51.67 (21.81)
FLR ratio increase^a^, %	12.73 (4.8)
Kinetic growth rate^a^, %/week	2.98 (1.29)

^a^Data available for 37 patients

*SD* Standard Deviation

### Association of factors with FRL increase after PVE

**Fig. 4:**
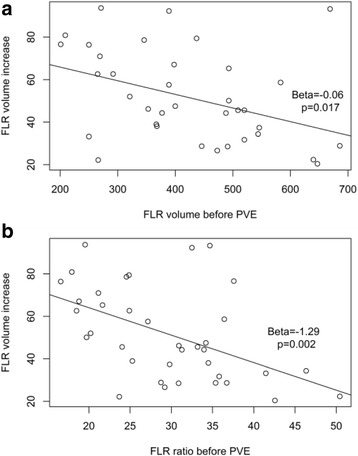
Graph showing small FLR superior growth. Graph showing the concept that smaller FLRs will grow most after PVE through the negative association observed between FLR volume (*top*) and FLR/TFLV ratio (*bottom*) and increase in the FLR after PVE

A negative association was observed between FLR volume and increase in the FLR after PVE (Beta = −0.06, *P* = 0.017, Fig. 4 top) and between the FLR/TFLV ratio before PVE and the increase in the FLR after PVE (Beta = −1.29, *P* = 0.002, Fig. 4 bottom).

### Complications

**Fig. 5:**
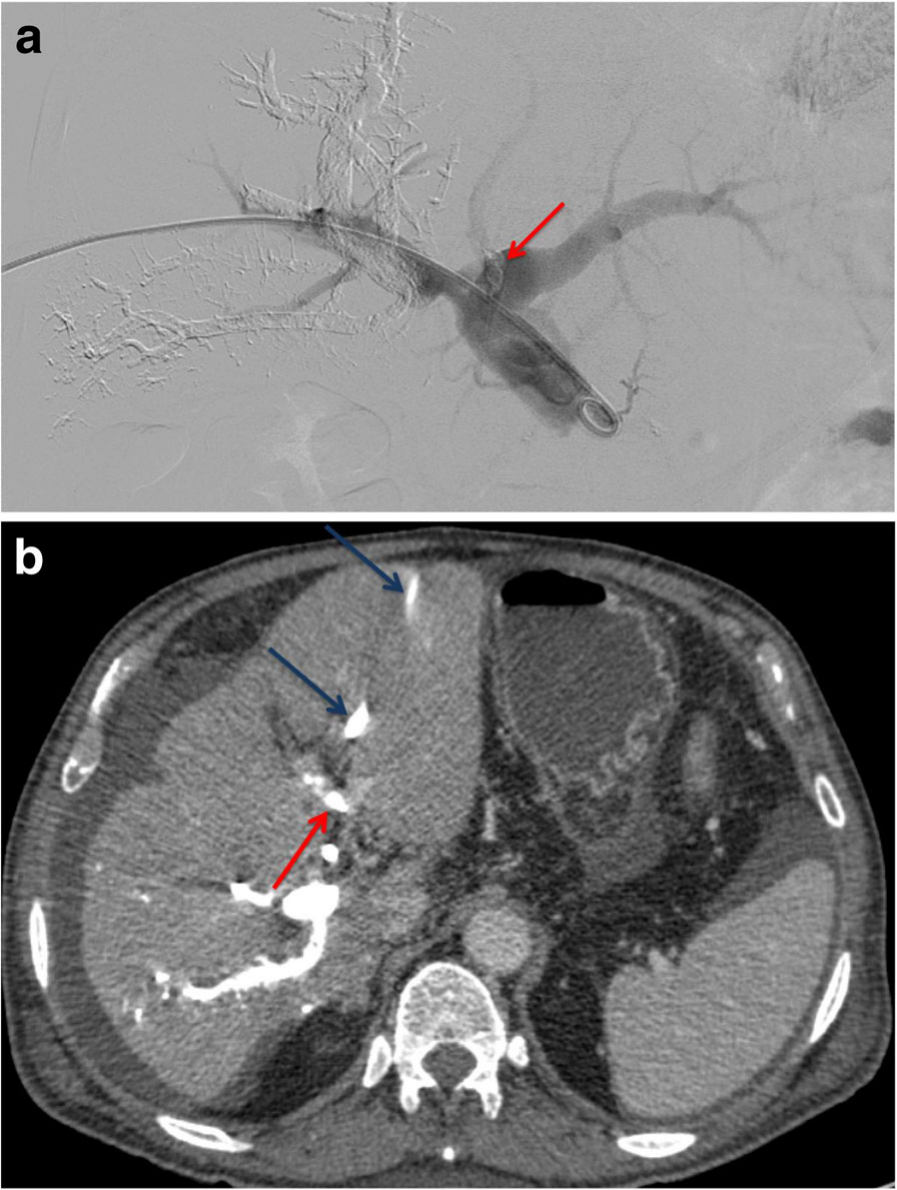
**a** Portography showing the dislodged NBCA. Direct portography during PVE showing the dislodged NBCA fragment in the left portal vein (red arrow). **b** CT showing dislodged NBCA. Contrasted-enhanced CT 4 weeks after PVE showing the NBCA fragment in the left portal vein (red arrow). This patient presented a 20% FLR hypertrophy but deceased due to fulminant cholangitis before surgery. (dark-blue arrow - Biliary drain trajectory in the liver)

Of the 50 patients submitted to 52 PVE procedures three experienced major complications (5.7%): significant migration of NBCA fragments to the FLR (*n* = 1), a subcapsular biloma (*n* = 1) and FLR portal vein stenosis due to NBCA fragment dislodgment associated with cholangitis (*n* = 1) and. The latter patient was a 74 year-old male patient with cholangiocarcinoma submitted to percutaneous biliary drainage and PVE at the same time. During catheter manipulation there was glue dislodgment to the main left portal vein creating a stenosis. This patient showed the lowest rate of FLR increase in our study (20%). He was re-admitted to the hospital 25 days after PVE with cholangitis and deceased 7 days afterwards due to refractory sepsis. Solely in this patient the complication precluded liver surgery (Fig. 5a and b).

Nine patients presented incidental findings or adverse events: 5 cases of very mild and minimal NBCA migration to the non-embolized liver, 1 case of small NBCA fragment migration to the right hepatic vein and 3 cases of nauseas and vomiting. All these patients with minute fragments of glue in the FLR or in the right hepatic vein presented satisfactory hypertrophy levels probably secondary to their very small size and non-occlusive arrangement [15].

### Complications and the use of microcatheters

There were no major complications, regarding NBCA, in patients where PVE was achieved with a microcatheter. Moreover incidental findings and adverse events were much more common in patients that a microcatheter was not used (*n* = 7) than in patients in whom it was adopted (*n* = 2). This analysis did not reach statistical significance (*p* = 0.189).

### Surgical outcomes

Thirty-four patients were taken to the operating room although 3 patients presented intraoperative disease progression that prohibited liver resection. Thirty-onepatients eventually were submitted to hepatic surgery (62%). The executed liver resection procedures were as follows: right hepatectomy in 19 patients, right hepatectomy extended to segment IV in 8 patients, right hepatectomy extended to segment IV with resection of the caudate lobe and left portal vein reconstruction in 1 patient, resection of segments VI and VII in 2 patients and Associating Liver Partition and Portal vein Ligation for Staged hepatectomy (ALPPS) in 1 patient. Post hepatectomy complications comprised bile leak or fistula or biloma (*n* = 3), pneumonia (*n* = 1), intraoperative hepatic bleeding (n = 3), sepsis (n = 1), subcapsular abscess (n = 1) and liver insufficiency (n = 1). Hospital stay was 9.97 days on average (range 3 to 56 days). Three patients needed blood transfusion. Thirteen patients eventually died (four patientswithin 30 days of hepatectomy - three from liver hemorrhage and one from a severe pneumonia). Sixteen patients were not taken to surgery due to disease progression (*n* = 14), cholangitis, liver insufficiency and death (n = 1) and uncontrolled comorbidities (n = 1).

## Discussion

Liver surgery has certainly evolved in the last decades [5, 10] and for patients with less than adequate FLR volume before hepatic resections PVE is the procedure of choice in most hepatobiliary treatment centers [31]. Different PVE techniques and approaches have been described and numerous embolization materials have been tested to stimulate remnant liver growth [3, 13, 32]. Analyses of the induced hypertrophy of PVE with NBCA using animaland afterwards human subjects showed its greater capacity compared to that of PVE with other embolic materials [14, 32] that might be related to the provoked periportal inflammatory response [4, 32]. Patients in the present study presented a substantial degree of FLR hypertrophy and increase in the FLR/TFLV ratio in accordance with previously published data including studies that compared NBCA glue with microparticles and coils [14, 22, 33]. The mean absolute FLR volume increase of 52% and the FLR/TFLV ratio expansion of 12% are superior to the published hypertrophy results in studies using other embolic agents than NBCA [14, 21, 34, 35]. Previously reported figures for PVE using NBCA, polyvinyl alcohol particles (PVA) plus coils/vascular plugs, gelatin sponge, PVA alone and fibrin glue are in the range of 47%-79%, 24%-54%, 17%-37%, 24%-32% and 27%-31% of FLR volume increase respectively [4, 24, 36, 37].

Another important and very discussed technical point in PVE is which side should be elected for the puncture, the contralateral, achieved through an access of the FLR’s peripheral portal branch, or the ipsilateral, attained by the puncture of the portal vein that will be removed in the near future hepatectomy. In the contralateral approach, developed in France [15, 38, 39], there is a potentially easier catheterization of the right portal subdivisions and the possibility to use smaller catheters [15] with the drawbackof potential harm to the FLR’s portal vein. The ipsilateral approach, in contrast, avoids puncture of the FLR but has a trickier catheterization [40]. In a systematic review that included studies from 1990 to 2011 [33], the ipsilateral approach accounted for 55% (*n* = 963) of all PVE procedures but in only 3% (28 procedures) the glue-lipiodol mixture (without coils nor particles nor amplatzer plug) was adopted as their sole embolic material. Moreover, the authors from this systematic review argued that it would be hard to manipulate glue from the ipsilateral side. Albeit we agree that PVE with glue requires extra caution, is a technically demanding procedure [24], and has a steep learning curve, the present study suggests that it can be performed through an ipsilateral approach. To our knowledge, this is one of the largest PVE series employing solely NBCA-lipiodol mixture as the embolic material through an ipsilateral approach.

These results show that the initial FLR volume and FLR/TFLV ratio were predictive factors for FLR hypertrophy after PVE, indicating that patients with lesser FLR volume or minor FLR/TFLV ratios at presentation will be the ones we can expect the greatest FLR enlargement. This association has been previously described in a study analyzing predictors of hypertrophy of the FLR after PVE in a non-cirrhotic population [24]. In cirrhotic patients with primary liver tumors this analysis was also performed and likewise similar results were shown [41, 42]. This correlation was also demonstrated in the surgical series that scrutinized liver regeneration and influencing factors. The general conclusion is that liver regeneration rate after resection is proportional to the volume of hepatic parenchyma removed at surgery whether or not liver dysfunction is present [43, 44]. As far as expectations and indications go for PVE these findings have direct influence in its daily practice. Since the degree of hypertrophy is inversely associated with the initial FRL volume, one should reaffirm PVE indication even for very small FLR volumes [24].

Of all complications associated with PVE recorded in our sample, only one precluded future liver surgery. This latter patient presented a stenosis of the left portal vein through the dislodgment of a glue fragment ensued near the completion of PVE and deceased 32 days after that from cholangitis and liver insufficiency. In this case we could have tried to pull the NBCA fragment back into the right portal vein as shown elsewhere [45], even though at that moment we did not have the appropriated material. While it is not stated in PVE guidelines or quality improvement statements [26] we currently maintain adequate retrieval materials such as snares and angioplasty balloons at our interventional radiology department, principally when dealing with embolic materials such as glue. It is also advisable to get satisfactory bilirubin clearance before PVE instead of performing both percutaneous biliary drainage and PVE procedures at the same time [29] as recommended in guidelines [26]. Particular attention should be devoted to avoid FLR glue migration since it can preclude liver surgery. When comparing complication rates between the groups with and without additional use of microcatheters there were no cases of major NBCA migration or dislodgment with its use and it was suggested that glue migration might be avoided with this coaxial technique. This difference did not reach statistical significance probably due to the small sample size. Additionally, the interpretation of this finding also suffers from the allocation of the use of the microcatheter, which was not random. We believe the use of microcatheters associated with the administration of small glue aliquots (e.g. 0.3 mL) in between abundant flushing with dextran or glucose 5% [26], is highly advisable when performing PVE with NBCA through an ipsilateral approach. Technical advises have been systematically addressed in the PVE NBCA publications, alerting for the extra care towards the end of the procedure where most of the targeted portal branches are already occluded, the few branches left usually demonstrate slow flow and complications, particularly embolic material reflux to the left portal vein, might occur [15].

The technical success rate (98%) and the clinical success rate of PVE (96%) in the present study were high and in accordance with the previous reported results [33]. Two patients (4%) presented insufficient hypertrophy after PVE and one of them could not be submitted to the planned surgery because of the associated development cholangitis and liver insufficiency. The other patient had a FLR before PVE which accounted for 17% of the TFLV and despite the significant hypertrophy of 76% and an increase to 30% in the FLR/TFLV ratio the liver surgeon decided to perform an ALPPS procedure to amplify FLR growth. The ALPPS was performed in May 2014 and this patient is currently on follow-up with no evidence of cancer related disease. In this series thirty-one patients (62%) were submitted to liver resections. One patient presented signs of liver failure within 30 days from hepatectomy with posterior convalescence. Out of the 31 patients submitted to liver surgery 18 (58%) are alive with no signs of hepatic insufficiency. Our 62% liver resection rate is below the usual published statistics for PVE in which approximately 70% to 80% of the originally planned liver resections after PVE are performed [33, 46]. In our group the vast majority of patients were not taken to liver resection after PVE due to disease progression. While this is the most frequent cause of not performing hepatectomies after PVE [33, 47], some of our cohort patients also suffered from inadequate long waiting periods for surgery after PVE due to local institutional impairments which might influence disease progression. Besides that, during PVE-induced liver regeneration, disease progression may be secondary to undetectable pre-existing tumor growth, and PVE may, consequently, perform as a surrogate marker of cancer biology, removing patients that are not suitable for surgery [46]. Notwithstanding tumor progression, FLR growth following PVE would have been sufficient to permit the planned hepatic resection in all but one of these patients.

This study has limitations. This was a retrospective study occurring over a period of five years and as such it is plausible that the improved patient selection for PVE and radiologists’ experience gained over the years could result in better outcomes. The data collection and extraction relied primarily on medical charts. The small sample hindered us from exploring other factors that might be linked to greater FLR increase. A strength of this study was the relative homogeneous patient population conceded only by non-cirrhotic patients which otherwise could have confused our hypertrophy results because of cirrhosis regeneration recognized variances [48]. Furthermore, even though NBCA is one of the main embolic agents used worldwide for PVE and it is suggested that it induces the highest FLR growth, its administration has been reported almost exclusively from the contralateral side. This study was able to show that it is possible to use NBCA for PVE without approaching the FLR.

## Conclusions

This study suggests that PVE with NBCA through an ipsilateral puncture is an effective procedure to permit major hepatectomies in patients with a small FLR. Meticulous attention is needed especially near the end of the embolization procedure to avoid complications.

## Abbreviations

ALPPS: Associating Liver Partition and Portal vein Ligation for Staged hepatectomy
CT: Computed tomography
FLR: Future liver remnant
KGR: Kineticgrowthrate
NBCA: n-butyl-cyanoacrylate
PVE: Portal vein embolization
TFLV: Total functional liver volume

## References

[1] Kinoshita H, Sakai K, Hirohashi K, Igawa S, Yamasaki O, Kubo S (1986). Preoperative portal vein embolization for hepatocellular carcinoma. World J Surg.

[2] Aoki T, Kubota K (2016). Preoperative portal vein embolization for hepatocellular carcinoma: consensus and controversy. World J Hepatol.

[3] Abdalla EK, Hicks ME, Vauthey JN (2001). Portal vein embolization: rationale, technique and future prospects. Br J Surg.

[4] de Baere T, Roche A, Elias D, Lasser P, Lagrange C, Bousson V (1996). Preoperative portal vein embolization for extension of hepatectomy indications. Hepatology.

[5] Azoulay D, Castaing D, Smail A, Adam R, Cailliez V, Laurent A, Lemoine A, Bismuth H (2000). Resection of nonresectable liver metastases from colorectal cancer after percutaneous portal vein embolization. Ann Surg.

[6] Kubota K, Makuuchi M, Kusaka K, Kobayashi T, Miki K, Hasegawa K, Harihara Y, Takayama T (1997). Measurement of liver volume and hepatic functional reserve as a guide to decision-making in resectional surgery for hepatic tumors. Hepatology.

[7] Starzl TE, Francavilla A, Halgrimson CG, Francavilla FR, Porter KA, Brown TH, Putnam CW (1973). The origin, hormonal nature, and action of hepatotrophic substances in portal venous blood. Surg Gynecol Obstet.

[8] Jaeck D, Bachellier P, Guiguet M, Boudjema K, Vaillant JC, Balladur P, Nordlinger B (1997). Long-term survival following resection of colorectal hepatic metastases. Association Française de Chirurgie. Br J Surg.

[9] Jaeck D, Oussoultzoglou E, Rosso E, Greget M, Weber JC, Bachellier P (2004). A two-stage hepatectomy procedure combined with portal vein embolization to achieve curative resection for initially unresectable multiple and bilobar colorectal liver metastases. Ann Surg.

[10] Adam R, Delvart V, Pascal G, Valeanu A, Castaing D, Azoulay D, Giacchetti S, Paule B, Kunstlinger F, Ghémard O, Levi F, Bismuth H (2004). Rescue surgery for unresectable colorectal liver metastases downstaged by chemotherapy: a model to predict long-term survival. Ann Surg.

[11] May BJ, Talenfeld AD, Madoff DC (2013). Update on portal vein embolization: evidence-based outcomes, controversies, and novel strategies. J Vasc Interv Radiol.

[12] Madoff DC, Abdalla EK, Vauthey JN (2005). Portal vein embolization in preparation for major hepatic resection: evolution of a new standard of care. J Vasc Interv Radiol.

[13] de Baere T, Denys A, Madoff DC (2007). Preoperative portal vein embolization: indications and technical considerations. Tech Vasc Interv Radiol.

[14] Guiu B, Bize P, Gunthern D, Demartines N, Halkic N, Denys A (2013). Portal vein embolization before right hepatectomy: improved results using n-butyl-cyanoacrylate compared to microparticles plus coils. Cardiovasc Intervent Radiol.

[15] Di Stefano DR, de Baere T, Denys A, Hakime A, Gorin G, Gillet M, Saric J, Trillaud H, Petit P, Bartoli JM, Elias D, Delpero JR (2005). Preoperative percutaneous portal vein embolization: evaluation of adverse events in 188 patients. Radiology.

[16] Madoff DC, Abdalla EK, Gupta S, Wu TT, Morris JS, Denys A, Wallace MJ, Morello FA, Ahrar K, Murthy R, Lunagomez S, Hicks ME, Vauthey JN (2005). Transhepatic ipsilateral right portal vein embolization extended to segment IV: improving hypertrophy and resection outcomes with spherical particles and coils. J Vasc Interv Radiol.

[17] Madoff DC, Hicks ME, Abdalla EK, Morris JS, Vauthey JN (2003). Portal vein embolization with polyvinyl alcohol particles and coils in preparation for major liver resection for hepatobiliary malignancy: safety and effectiveness--study in 26 patients. Radiology.

[18] Nagino M, Kamiya J, Nishio H, Ebata T, Arai T, Nimura Y (2006). Two hundred forty consecutive portal vein embolizations before extended hepatectomy for biliary cancer: surgical outcome and long-term follow-up. Ann Surg.

[19] Hong YK, Choi SB, Lee KH, Park SW, Park YN, Choi JS, Lee WJ, Chung JB, Kim KS (2011). The efficacy of portal vein embolization prior to right extended hemihepatectomy for hilar cholangiocellular carcinoma: a retrospective cohort study. Eur J Surg Oncol.

[20] Mise Y, Passot G, Wang X, Chen HC, Wei S, Brudvik KW, Aloia TA, Conrad C, Huang SY, Vauthey JN (2016). A Nomogram to predict hypertrophy of liver segments 2 and 3 after right portal vein Embolization. J Gastrointest Surg.

[21] Libicher M, Herbrik M, Stippel D, Poggenborg J, Bovenschulte H, Schwabe H (2010). Portal vein embolization using the amplatzer vascular plug II: preliminary results. Rofo.

[22] Jaberi A, Toor SS, Rajan DK, Mironov O, Kachura JR, Cleary SP, Smoot R, Tremblay St-Germain A, Tan K (2016). Comparison of clinical outcomes following glue versus polyvinyl alcohol portal vein Embolization for hypertrophy of the future liver remnant prior to right hepatectomy. J Vasc Interv Radiol.

[23] Nagino M, Nimura Y, Kamiya J, Kondo S, Kanai M (1996). Selective percutaneous transhepatic embolization of the portal vein in preparation for extensive liver resection: the ipsilateral approach. Radiology.

[24] de Baere T, Teriitehau C, Deschamps F, Catherine L, Rao P, Hakime A, Auperin A, Goere D, Elias D, Hechelhammer L (2010). Predictive factors for hypertrophy of the future remnant liver after selective portal vein embolization. Ann Surg Oncol.

[25] van der Vorst JR, van Dam RM, van Stiphout RS, van den Broek MA, Hollander IH, Kessels AG, Dejong CH (2010). Virtual liver resection and volumetric analysis of the future liver remnant using open source image processing software. World J Surg.

[26] Denys A, Bize P, Demartines N, Deschamps F, De Baere T, Europe, C. a. I. R. S. o (2010). Quality improvement for portal vein embolization. Cardiovasc Intervent Radiol.

[27] Shindoh J, Truty MJ, Aloia TA, Curley SA, Zimmitti G, Huang SY, Mahvash A, Gupta S, Wallace MJ, Vauthey JN (2013). Kinetic growth rate after portal vein embolization predicts posthepatectomy outcomes: toward zero liver-related mortality in patients with colorectal liver metastases and small future liver remnant. J Am Coll Surg.

[28] Goldberg SN, Grassi CJ, Cardella JF, Charboneau JW, Dodd GD, Dupuy DE, Gervais D, Gillams AR, Kane RA, Lee FT, Livraghi T, McGahan J, Phillips DA, Rhim H, Silverman SG, Committee, S. o. I. R. T. A, Ablation, I. W. G. o. I.-G. T (2005). Image-guided tumor ablation: standardization of terminology and reporting criteria. Radiology.

[29] Guiu B, Bize P, Demartines N, Lesurtel M, Denys A (2014). Simultaneous biliary drainage and portal vein embolization before extended hepatectomy for hilar cholangiocarcinoma: preliminary experience. Cardiovasc Intervent Radiol.

[30] Lee EC, Park SJ, Han SS, Park HM, Lee SD, Kim SH, Lee IJ, Kim HB (2017). Mortality after portal vein embolization: two case reports. Medicine (Baltimore).

[31] Adams RB, Haller DG, Roh MS (2006). Improving resectability of hepatic colorectal metastases: expert consensus statement by Abdalla et al. Ann Surg Oncol.

[32] de Baere T, Denys A, Paradis V (2009). Comparison of four embolic materials for portal vein embolization: experimental study in pigs. Eur Radiol.

[33] van Lienden KP, van den Esschert JW, de Graaf W, Bipat S, Lameris JS, van Gulik TM, van Delden OM (2013). Portal vein embolization before liver resection: a systematic review. Cardiovasc Intervent Radiol.

[34] Covey AM, Brown KT, Jarnagin WR, Brody LA, Schwartz L, Tuorto S, Sofocleous CT, D'Angelica M, Getrajdman GI, DeMatteo R, Kemeny NE, Fong Y (2008). Combined portal vein embolization and neoadjuvant chemotherapy as a treatment strategy for resectable hepatic colorectal metastases. Ann Surg.

[35] van den Esschert JW, de Graaf W, van Lienden KP, Busch OR, Heger M, van Delden OM, Gouma DJ, Bennink RJ, Laméris JS, van Gulik TM (2009). Volumetric and functional recovery of the remnant liver after major liver resection with prior portal vein embolization : recovery after PVE and liver resection. J Gastrointest Surg.

[36] Giraudo G, Greget M, Oussoultzoglou E, Rosso E, Bachellier P, Jaeck D (2008). Preoperative contralateral portal vein embolization before major hepatic resection is a safe and efficient procedure: a large single institution experience. Surgery.

[37] Covey AM, Tuorto S, Brody LA, Sofocleous CT, Schubert J, von Tengg-Kobligk H, Getrajdman GI, Schwartz LH, Fong Y, Brown KT (2005). Safety and efficacy of preoperative portal vein embolization with polyvinyl alcohol in 58 patients with liver metastases. AJR Am J Roentgenol.

[38] de Baere T, Roche A, Vavasseur D, Therasse E, Indushekar S, Elias D, Bognel C (1993). Portal vein embolization: utility for inducing left hepatic lobe hypertrophy before surgery. Radiology.

[39] Azoulay D, Castaing D, Krissat J, Smail A, Hargreaves GM, Lemoine A, Emile JF, Bismuth H (2000). Percutaneous portal vein embolization increases the feasibility and safety of major liver resection for hepatocellular carcinoma in injured liver. Ann Surg.

[40] Denys A, Prior J, Bize P, Duran R, De Baere T, Halkic N, Demartines N (2012). Portal vein embolization: what do we know?. Cardiovasc Intervent Radiol.

[41] Imamura H, Shimada R, Kubota M, Matsuyama Y, Nakayama A, Miyagawa S, Makuuchi M, Kawasaki S (1999). Preoperative portal vein embolization: an audit of 84 patients. Hepatology.

[42] Denys A, Lacombe C, Schneider F, Madoff DC, Doenz F, Qanadli SD, Halkic N, Sauvanet A, Vilgrain V, Schnyder P (2005). Portal vein embolization with N-butyl cyanoacrylate before partial hepatectomy in patients with hepatocellular carcinoma and underlying cirrhosis or advanced fibrosis. J Vasc Interv Radiol.

[43] Tani M, Tomiya T, Yamada S, Hayashi S, Yahata K, Tamura Y, Akiyama M, Kawai S, Masaki N, Fujiwara K (1994). Regulating factors of liver regeneration after hepatectomy. Cancer Chemother Pharmacol.

[44] Nagino M, Ando M, Kamiya J, Uesaka K, Sano T, Nimura Y (2001). Liver regeneration after major hepatectomy for biliary cancer. Br J Surg.

[45] Dobrocky T, Kettenbach J, Lopez-Benitez R, Kara L (2015). Disastrous portal vein Embolization turned into a successful intervention. Cardiovasc Intervent Radiol.

[46] Ironside N, Bell R, Bartlett A, McCall J, Powell J, Pandanaboyana S (2017). Systematic review of perioperative and survival outcomes of liver resections with and without preoperative portal vein embolization for colorectal metastases. HPB (Oxford).

[47] Pamecha V, Glantzounis G, Davies N, Fusai G, Sharma D, Davidson B (2009). Long-term survival and disease recurrence following portal vein embolisation prior to major hepatectomy for colorectal metastases. Ann Surg Oncol.

[48] Yamanaka N, Okamoto E, Kawamura E, Kato T, Oriyama T, Fujimoto J, Furukawa K, Tanaka T, Tomoda F, Tanaka W (1993). Dynamics of normal and injured human liver regeneration after hepatectomy as assessed on the basis of computed tomography and liver function. Hepatology.

